# Orosomucoid 1 promotes epirubicin resistance in breast cancer by upregulating the expression of matrix metalloproteinases 2 and 9

**DOI:** 10.1080/21655979.2021.1987067

**Published:** 2021-10-27

**Authors:** Luo Qiong, Jun Yin

**Affiliations:** Department of Breast and Thyroid Surgery, Affiliated Hengyang Hospital, Southern Medical University (Hengyang Central Hospital), Hengyang, People’s Republic of China

**Keywords:** Orosomucoid 1 (ORM1), epirubicin, matrix metalloproteinases-2 (MMP-2), matrix metalloproteinases-9 (MMP-9), AKT, Erk, breast cancer

## Abstract

Orosomucoid 1 (ORM1) has been shown to be upregulated in the serum of breast cancer patients; however, the expression and function of ORM1 in breast cancer remains unknown. We measured the expression of ORM1 in breast cancer tissues and cell lines using qRT-PCR. A colony formation assay was done to assess cell proliferation and Transwell and wound healing assays were performed to determine the migration and invasion capacity of the cells, respectively. In addition, a CCK-8 assay was used to measure epirubicin cytotoxicity and western blot assays were done to analyze the putative mechanisms of epirubicin sensitivity. We found that the expression of ORM1 was upregulated in breast cancer tissues and cell lines. The expression of ORM1 enhanced the proliferation and migration of the cell lines. In contrast, down-regulation of ORM1 inhibited the expression of MMP-2 and MMP-9 and activation of the AKT/ERK signaling pathway. Therefore, ORM1 may represent a potential therapeutic target for breast cancer and promote epirubicin resistance by regulating the expression of MMP-2 and MMP-9, as well as activating the AKT/ERK signaling pathway.

## 1. Introduction

Breast cancer is a tumor type with the highest incidence in women [1]. According to the American Cancer Statistics Report on Breast cancer in 2021, new cases accounted for 30% of all female malignant tumors, ranking at the top in incidence for female malignant tumors [2]. In China, breast cancer tends to occur in a younger population, it endangers women’s health, and is difficult to eradicate [3]. Moreover, a small percentage of men also develop breast cancer accounting for 2,000 cases each year in the United States [4]. Currently, there are no effective preventative measures for breast cancer. Surgery and drug treatment yield curative effects in early stage breast cancer; however, most patients have developed tumor metastasis at the time of diagnosis. Thus, early diagnosis directly affects the treatment and prognosis of this tumor type [5,6].

Targeted therapy based on the identification of new oncogenes and tumor targets has also played a key role in breast cancer treatment [7,8]. With the introduction of the concept of molecular typing and precision medicine, breast cancer research is gradually changing from evidence-based empirical treatment to individualized treatment based on whole genome sequencing and gene mutation data. Therefore, identifying new breast cancer genes, discovering their regulatory mechanisms, biological effects, and clinical relevance have become the goal of breast cancer research in recent years. Most importantly, evaluating new molecular targets for drug therapy is imperative [9].

Alpha-acid glycoprotein or orosomucoid (AGP or ORM) is an important muscle relaxant binding protein. Polymorphisms in this gene result in a variation in the effects of muscle relaxation over time [10]. ORM consists of 183 amino acids with a molecular weight of 40 kDa. It is synthesized by hepatocytes and secreted into the blood. There are two ORM subtypes, ORM1 and ORM2, which are encoded by two closely linked genes on chromosome 9 with a length of 11.5 KB. The synthesis of ORM is controlled by different alleles at two loci, ORM1 and ORM2 [11]. The difference between ORM1 and ORM2 is only 22 bases. ORM is an acute phase reaction protein and its expression is increased by infection, allergy, burns, breast cancer, lung cancer, and chronic nephritis. The expression of ORM1 and ORM2 is dysregulated in many malignant cancers. For example, compared with normal liver tissue, the expression of ORM1 and ORM2 are both downregulated in liver tumors [12]. In breast cancer, the level of serum ORM in breast cancer patients is higher compared with that in healthy women [13]. However, the function of ORM1 in breast cancer remains unknown.

Chemotherapy is important for the treatment of breast cancer, whereas drug resistance is the primary reason for the failure of chemotherapy. ORM1 has been shown to play an important role in chemotherapeutic drug resistance in breast cancer cells. Therefore, we hypothesize that ORM1 affects the sensitivity of epirubicin-resistant cancer cells. In this study, we evaluated ORM1 expression in epirubicin-resistant breast cancer cells and determined the underlying mechanism of action of ORM1. The data indicate that ORM1 expression is increased in both breast cancer tissues and cell lines, and upregulation of ORM1 promotes proliferation, migration, and resistance to epirubicin though increased expression of matrix metalloproteinases 2 (MMP-2) and 9 (MMP-9) and activation of the AKT/ERK signaling pathway.

## 2. Materials and methods

### 2.1. Patient tissues

A total of 10 breast cancer tissues were collected along with 10 matched adjacent normal tissue samples (the sampling site was at least 2 cm away from the boundary of the tumors). The fresh tissue samples were collected immediately after tumor resection and cryopreserved in liquid nitrogen. Patients had not received any treatment including neoadjuvant radiotherapy, chemotherapy, or traditional Chinese medicine prior to collection. The patients had no other malignant tumors and all specimens were obtained with the approval of the medical ethics committee of the Affiliated Hengyang Hospital of the Southern Medical University. Informed consent was obtained from all of the patients (Ethical Approval Number: 023).

### 2.2. Immunohistochemical (IHC)

Protein expression was determined by an Elivision two-step immunohistochemical method. Tissue samples were paraffin-embedded and sectioned. The paraffin sections were dried for 2 hours, dewaxed, and washed with PBS three times for 3 minutes each. The slides were added to citrate buffer and the antigens were retrieved using a microwave. After incubation with 3% H_2_O_2_ at room temperature for 10 min, the slides were rinsed three times with PBS. The corresponding primary antibody (diluted 1:200, Proteintech) was added and incubated at room temperature for 2 hours and the slides were washed 3 times with PBS. A polymer reinforcer was added dropwise and incubated at room temperature for 20 min. Rabbit anti-ORM1 polyclonal antibody (diluted 1:200, Proteintech) was added and incubated at room temperature for 30 min. After incubating with DAB solution, the slides were observed by microscopy. They were counterstained with hematoxylin, differentiated with 0.1% HCl, washed with tap water, and cyanated. The slices were dehydrated, dried with gradient alcohol, washed with xylene, sealed with neutral gum, dried, and observed by microscopy.

### 2.3. Cell culture

Three cell lines, HBL-100, MDA-MB-231, and MDA-MB-231/EPI, were purchased from the Procell Life Science Co. Cells were cultured with DMEM/F12 medium (Invitrogen, Carlsbad, CA, USA) containing 10% FBS (Invitrogen, Carlsbad, CA, USA) at 37°C in a 5% CO_2_ incubator (thermo, mass., USA).

### 2.4. qRT-PCR

Total RNA was isolated using Trizol (Vazyme, Nanjing, China) and cDNA was synthesized using the GoScript Reverse Transcription System (Promega, Madison, WI, USA). Relative RNA expression levels were measured by quantitative real-time PCR (qPCR) using the GoTaq qPCR Master Mix (Promega, Madison, WI, USA). GAPDH was used as an internal control. The relative levels of RNA were calculated by the 2− ΔΔCt method. The sequences of the gene-specific primers used are listed in Table 1.

**Table 1.** The primer sequences

Gene	Primer sequences	
ORM1	Forward	CTGACAAGCCAGAGACGACCAA
	Reverse	TGCTTCTCCAGTGGCTCACACT
GAPDH	Forward	GTCTCCTCTGACTTCAACAGCG
	Reverse	ACCACCCTGTTGCTGTAGCCAA

### 2.5. Cell transfection

Small interfering RNAs (siRNAs) were purchased from RiboBio (Guangzhou, China). The pcDNA3-MMP2, pcDNA3-MMP9, and pcDNA3 vector plasmids were obtained from CUSABIO (Wuhan, China). Transfection of the siRNAs and plasmids was done using Lipofectamine 3000 (PolyPlus-transfection, France). Cells were divided into different groups as follows: (1) Blank: untransfected cells; (2) si-NC: cells incubated with control siRNA; (3) si-ORM1: cells incubated with si-ORM1; (4) Vector: cells incubated with pcDNA3 vector; (5) OE-MMP2/MMP9: cells incubated with pcDNA3-MMP2 or pcDNA3-MMP9. The transfections were performed when cells reached 70%–80% confluence, and RNA and protein were harvested after 48 h.

### 2.6. Colony formation assay

For the colony formation assay, 300 cells were seeded into a 12-well dish and allowed to grow until colonies were visible (10–14 days). The colonies were first washed with PBS, fixed with 4% paraformaldehyde, and stained with crystal violet. Cells were counted under a microscope (PRECISE, Beijing, China). The colony number was estimated using Image J software.

### 2.7. Transwell assay

After the cells were digested with trypsin, the cells in each group were resuspended in serum-free medium. The cell suspension was adjusted to a density of 4 × 10^5^ cells/mL. A sterile Transwell chamber was placed into a 24-well plate and 100 *μ*L of cell suspension was seeded into the upper compartment, whereas 800 *μ*l of complete medium containing 10% FBS was added to the lower compartment. The cells were incubated at 37°C for an additional 18 h. The Transwell chamber was gently washed three times with 0.01 M PBS and fixed with 4% paraformaldehyde for 30 minutes. The cells were then stained with crystal violet solution for 20 min. Image J software was used to analyze the number of stained cells in the images and the number of cells in each field was counted.

### 2.8. In vitro cytotoxicity assays

The viability of the cells treated with various concentrations of epirubicin (0.5, 1.0, 1.5, and 2.0 *μ*M) was determined by the Cell Counting Kit-8 assay (CCK-8, 7Sea Biotech, Shanghai, China) as previously described [14]. Cells (5,000/well) were seeded into 96-well plates for 24 h and treated with epirubicin for 48 h. The half-maximum inhibitory concentration (IC_50_) was calculated by nonlinear regression analysis using GraphPad Prism 8.0 software (GraphPad Software, La Jolla, CA, USA).

### 2.9. Wound healing assay

The cells were seeded into 6-well plates at a concentration of 5 × 10^5^ cells/well and cultured in 5% CO_2_ incubator at 37°C until the cell confluence reached 95%–100%. A scratch was made on the 95%–100% fusion cell monolayer to form a cell-free area and PBS was used to wash away the loose cells. Cell growth inhibitors were added to the cultured cells. The cell-free areas at different time points were photographed and analyzed by Image J software. Approximately 6 to 8 horizontal lines were randomly drawn to calculate the mean of the distance between the cells in the cell-free area. The cell migration rate for each group was compared with the distance between the scratched areas.

### 2.10. Annexin V-PE/7-AAD Apoptosis assay

Cells were cultured with epirubicin (2 *μ*M) for 48 h, collected, and divided into two groups. One group was transfected with si-ORM1 and the other group was transfected with control siRNA, followed by incubation for 48 h. The annexin V-PE/7-AAD apoptosis kit (Vazyme, Nanjing, China) was used to analyze the cells. The cultured cells were collected into groups, digested with trypsin, and centrifuged. For washing, the supernatant was removed, PBS was added, the cells were resuspended, and the procedure was repeated 3 times. Next, 250 *μ*L of binding buffer was added to the resuspended cells. The cell suspension (100 *μ*L) was mixed with 5 *μ*L Annexin V-PE and 10 *μ*L 7-AAD solution, and incubated for 15 min in the dark. The stained cells were immediately analyzed by flow cytometry. Finally, flowjo software was used for data analysis. The mortality rate in the fourth quadrant was analyzed for each group and compared.

### 2.11. Western blot analysis

RIPA lysis was used to prepare cell extracts. After protein concentration was measured, the extracts were mixed with loading buffer and denatured by heating in a boiling water bath for 5 minutes. Electrophoresis was carried out at a voltage of 80 V for 30 min and increased to 120 V for 1–2 h after the bromophenol blue had entered the bottom of the gel. The separated proteins were transferred to membranes, rinsed in TBST for 5 minutes, and blocked with 5% BSA for 60 minutes at room temperature. The primary antibodies were as follows: ORM1 (1:1000, 66,097-1-Ig, Proteintech), AKT (1:1000, #4691S, Cell Signaling Technology), p-AKT (1:1000, # 4060S, Cell Signaling Technology), Erk (1:1000, #8544S, Cell Signaling Technology), p-Erk (1:1000, #4370S, Cell Signaling Technology), MMP-2 (1:1000, #40994S, Cell Signaling Technology), MMP-9 (1:1000, #13667S, Cell Signaling Technology), and GAPDH (1:5000, 60,004-1-Ig, USA).

### 2.12. Statistical analysis

All statistical analyses were done using SPSS version 20.0 and GraphPad Prism 8.0 Software. Data are presented as the$\bar{x}\pm$ standard error of the mean (SEM). Data were analyzed by a Student’s t-test for the comparison of two independent groups or one-way ANOVA for univariate comparisons. A Pearson coefficient was calculated for linear correlations between two different parameters. The statistical parameters are provided in the figure legends and *p*-values less than 0.05 were considered statistically significant. All experiments were repeated a minimum of three times.

## 3. Results

### 3.1. ORM1 expression is upregulated in breast cancer

**Figure 1.**
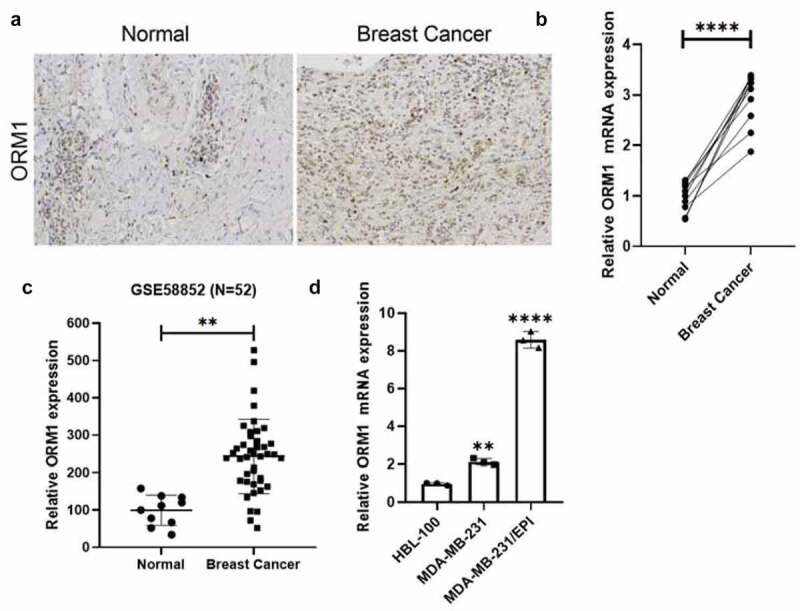
**The expression of ORM1 is upregulated in breast cancer** a IHC staining of the tissues derived from the patients. b The expression level of ORM1 mRNA in the breast cancer patients’ tissues and the adjacent normal tissues. Data are presented as mean ± SEM from three independent experiments, n = 10, respectively. **** *P* < 0.0001. c The expression of ORM1 was reanalyzed from the previously published dataset GSE58812. d The expression level of ORM1 mRNA in the normal breast cell line HBL-100 and the breast cancer cell lines. Data are presented as mean ± SEM from three independent experiments. ** *P* < 0.01; **** *P* < 0.0001, compared with the HBL-100 cell line

We collected 10 pairs of breast cancer specimens along with corresponding adjacent normal tissues from the pathology department of Hengyang Central hospital, and the expression of ORM1 was measured by IHC and qRT-PCR. As shown in Figure 1(a,b), compared with normal tissues, the expression of ORM1 mRNA was increased in breast cancer tissues. The upregulated expression of ORM1 in breast cancer cells was consistent with data from the reanalysis of the GSE58812 dataset (Figure 1(c)). Moreover, compared with the normal breast cell line HBL-100, the expression of ORM1 was increased in the MDA-MB-231 breast cancer cell line and the epirubicin-resistant MDA-MB-231/EPI cell line (Figure 1(d)). Taken together, the expression of ORM1 was upregulated both in breast cancer tissues and cell lines, which included an epirubicin-resistant cell line.

### 3.2. ORM1 promotes the proliferation, migration, and epirubicin resistance of breast cancer cells

**Figure 2.**
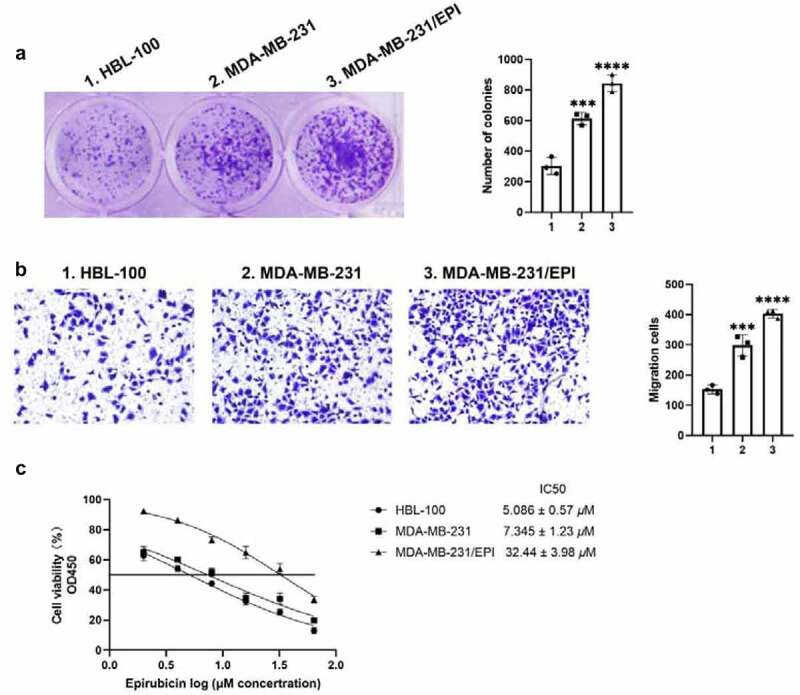
**ORM1 promote the proliferation, migration and epirubicin resistance of breast cancer cells** a Colony formation assay was performed to detect the proliferation of the cell lines, scale bar: 5 *μ*m. b Transwell assay was performed to detect the migration of the cell lines, scale bar: 50 *μ*m. c CCK-8 assay was performed to analyze the epirubicin IC50 values of the cell lines. d Data are presented as mean ± SEM from three independent experiments. *** *P* < 0.001; **** *P* < 0.0001, compared with the HBL-100 cell line

Cell proliferation and migration are important events in tumorigenesis and development. To determine the biological role of ORM1 in breast cancer, colony formation and Transwell assays were performed to analyze the proliferation and migration of the cell lines, respectively. As shown in Figure 2(a), compared with HBL-100 cells, the number of colonies was significantly higher in MDA-MB-231 cells and the epirubicin-resistant cell line, MDA-MB-231/EPI. The migration ability exhibited the same tendency. Compared with the HBL-100, the number of migrating cells was significantly higher in the MDA-MB-231 and MDA-MB-231/EPI cell lines. In addition, the CCK-8 assay was used to analyze the cytotoxicity of epirubicin in each cell line. As shown in Figure 2(c), MDA-MB-231/EPI exhibited the highest epirubicin IC_50_ values. Taken together, ORM1 is associated with increased proliferation, migration, and epirubicin resistance of breast cancer.

### 3.3. Downregulation of ORM1 suppresses the malignant phenotype of breast cancer cells

**Figure 3.**
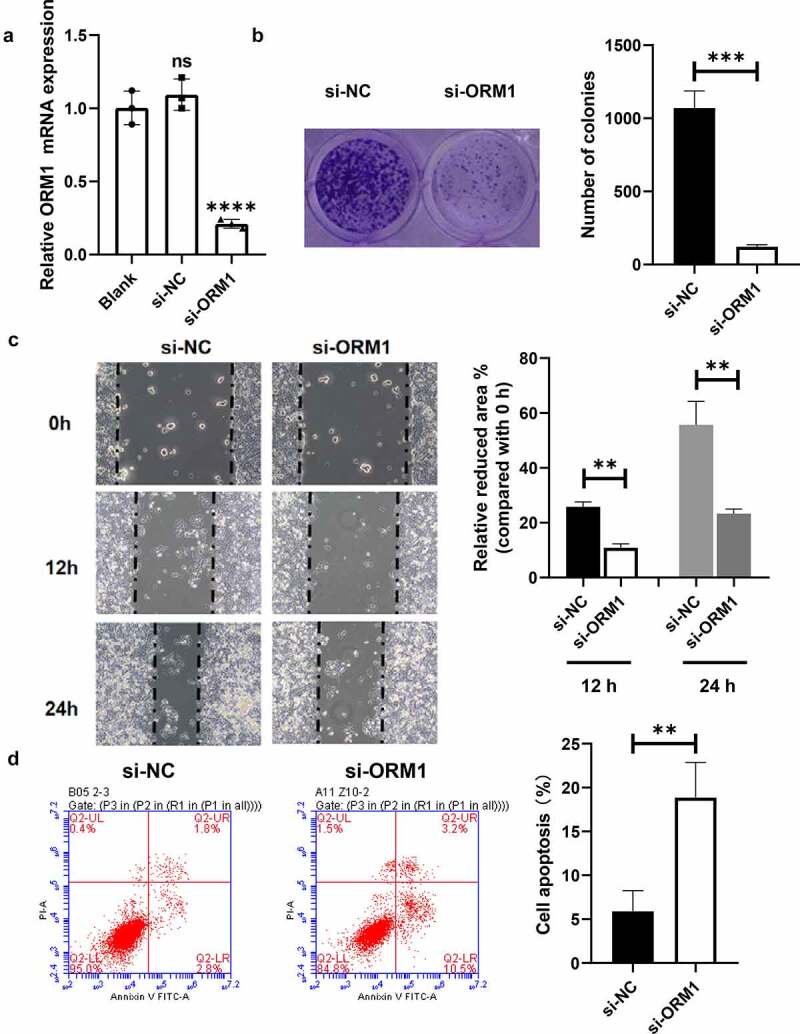
**Downregulation of ORM1 suppressed the malignant phenotype of breast cancer cells a** qRT-PCR assay was performed to detect the transfection efficiency on si-ORM1; b Colony formation was performed to detect the proliferation after downregulating ORM1, scale bar: 5 *μ*m; c Wound healing assay was performed to detect the migration at different time after downregulating ORM1; d Flow cytometry was performed to detect the apoptosis of cells treating with epirubicin after downregulating ORM1

Quantitative RT-PCR was used to measure the efficiency of transfection of siRNAs into MDA-MB-231/EPI cells. As shown in Figure 3(a), compared with the control, the mRNA levels of ORM1 in the si-NC group showed no significant difference, whereas the expression of ORM1 in the si-ORM1 group was significantly lower. A colony formation assay was performed to analyze the proliferation ability of the transfected breast cancer cells. As shown in Figure 3(b), compared with the si-NC cells, the number of colonies in the si-ORM1 group was significantly decreased. A wound healing assay was then performed to evaluate breast cancer cell migration (Figure 3(c)). After 12 and 24 hours of culture, the migration area of the cells in the si-ORM1 group was smaller compared with that of the si-NC group. In addition, flow cytometry was used to detect apoptosis in cells cultured with 2 mM epirubicin. As shown in Figure 3(d), compared with the si-NC group, the apoptosis rate of the si-ORM1 group was significantly increased. Taken together, downregulation of ORM1 suppresses the malignant phenotype and increases the drug sensitivity of epirubicin-resistant breast cancer cells.

Data are presented as mean ± SEM from three independent experiments. ns *P* > 0.05; ** *P* < 0.01; *** *P* < 0.001; **** *P* < 0.0001.

### 3.4. ORM1 suppresses the migration of breast cancer cells by targeting MMP-2 and MMP-9

**Figure 4.**
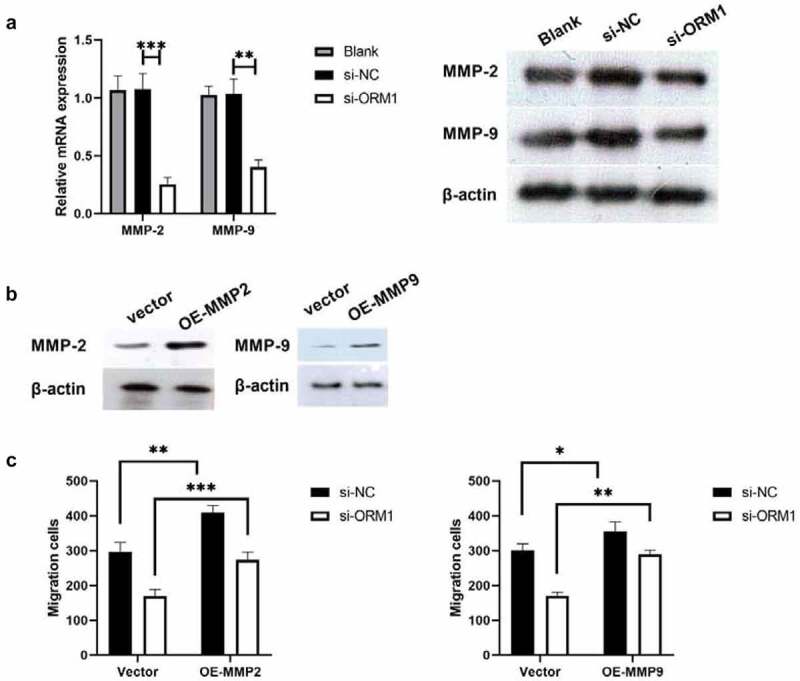
**ORM1 suppressed the migration of breast cancer cells via targeting MMP-2 and MMP-9** a -PCR assay and western blot were performed to detect the mRNA expression of MMP-2 and MMP-9 after downregulating ORM1; b Western blot was performed to detect the expression of MMP-2 and MMP-9 after upregulating MMP-2 or MMP-9; c Transwell assay was performed to detect the migration after upregulating MMP-2 and MMP-9, respectively

Thus far, the data from this study indicate that ORM1 may promote the malignant phenotype of breast cancer cells, so we further analyzed the underlying mechanism by western blot analysis. MMP-2 and MMP-9 are associated with epirubicin resistance in urothelial carcinoma (UC) [15]. Therefore, we used western blot analysis to measure the expression of MMP-2 and MMP-9 protein in MDA-MB-231/EPI cells after downregulating ORM1. As shown in Figure 4(a), compared with si-NC, the expression of MMP-2 and MMP-9 was significantly decreased in si-ORM1-transfected cells. We next investigated the underlying mechanism of this effect on breast cancer cell migration. As shown in Figure 4(b), the protein levels of MMP-2 and MMP-9 were significantly increased in the OE-MMP2 and OE-MMP9 group. Furthermore, compared with the si-ORM1+ Vector group, cell migration was increased in the si-ORM1+ OE-MMP2 and si-ORM1+ OE-MMP9 groups. Taken together, ORM1 restores the migration ability of breast cancer cells by targeting MMP-2 and MMP-9.

Data are presented as mean ± SEM from three independent experiments. ns *P* > 0.05; ** *P* < 0.01; *** *P* < 0.001; **** *P* < 0.0001.

### 3.5. ORM1 activates the AKT/ERK signaling pathway

**Figure 5.**
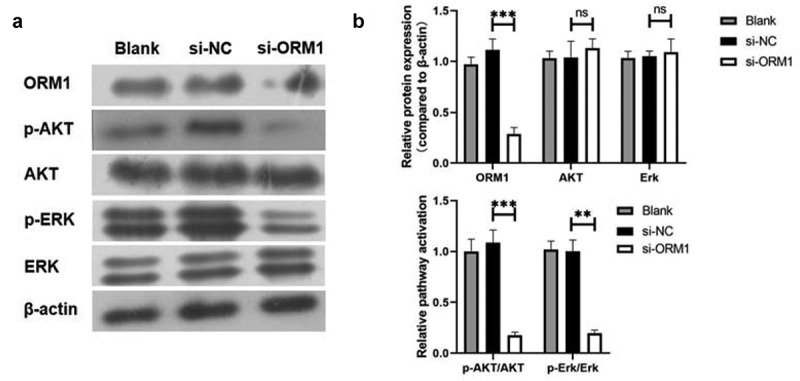
**ORM1 could upregulate the MMP-2 and MMP-9 then activate the AKT/Erk signaling pathways** A-B. Western blot assay was performed to detect the level of ORM1, p-AKT, AKT, p-Erk and Erk

We examined the activation of the AKT and ERK signaling pathways. As shown in Figure 5(a,b), compared with the si-NC group, both AKT and ERK levels showed no significant difference. However, compared with the si-NC group, p-AKT/AKT and p-Erk/Erk were significantly decreased in the si-ORM1 group. Taken together, ORM1 activates the AKT/ERK signaling pathway.

Data are presented as mean ± SEM from three independent experiments. ns *P* > 0.05; ** *P* < 0.01; *** *P* < 0.001.

## 4. Discussion

Breast cancer is a malignant tumor with the highest incidence and mortality in women [16]. The increased mortality rate of breast cancer patients is associated with the emergence of drug-resistance tumor cells [17,18]. The current treatment for breast cancer includes surgery, radiotherapy, chemotherapy, and immunotherapy [19,20]. Although some progress has been made, not only in the diagnosis, but also in treatment, the prognosis of breast cancer patients remains poor because of recurrence and metastasis following surgery. An improved understanding of the molecular mechanisms responsible for the pathogenesis and progression of breast cancer, and the discovery of key oncogenes that control development, metastasis, and invasion, are particularly important for the design of new strategies for the treatment of patients diagnosed with metastatic and recurrent breast cancer. Chemotherapy can effectively eliminate tumor cells and remains one of the most important treatment methods. However, because of the development of multidrug resistance, the five-year survival rate is low [17]. Therefore, it is urgent to seek more effective treatment strategies to improve treatment for breast cancer. In the present study, the data revealed that when compared with normal breast tissue and cell lines, the expression of ORM1 was upregulated in breast cancer tissues and cell lines. Moreover, the upregulation of ORM1 not only enhanced the proliferation, but also the migration of breast cancer cells. Furthermore, ORM1 expression was increased in an epirubicin-resistant cell line.

Among the causes of death from cancer, chemotherapeutic drug resistance accounts for a significant fraction [21]. In some cancer patients, intrinsic drug resistance occurs following first-line treatment. Data have shown that approximately 90% of cancer deaths are associated with drug resistance [22]. Moreover, multidrug resistance renders the subsequent treatment of tumors more difficult. Drug resistance exists in different types of breast cancer, although acquired resistance is the main problem. ORM generally binds to basic and neutral drugs, and recent studies demonstrated that some acidic drugs exhibit high affinity for ORM. In one study, a gastric cancer patient with poorly differentiated adenocarcinoma showed significant tolerance to atracurium during subtotal gastrectomy, which was related to increased serum ORM levels [23]. In a rat infection model, atracurium tolerance was related to increased levels of ORM in rats [24]. Albumin (ALB) is an important drug binding protein in the human body and combines with a variety of endogenous and exogenous substances, thus affecting their pharmacokinetics and pharmacodynamics [25]. The binding of ORM to epirubicin is even higher compared with that of albumin [26]. In the present study, we found that compared with the MDA-MB-231 cell line, the epirubicin-resistant cell line, MDA-MB-231/EPI, exhibited higher ORM1 expression and epirubicin IC_50_ values. Moreover, downregulating ORM1 using si-ORM1 promoted apoptosis following epirubicin treatment. Taken together, our results indicate that upregulation of ORM1 increases epirubicin resistance in breast cancer cells in vitro.

As two of the most widely studied matrix metalloproteinases (MMP), MMP-2 and MMP-9 play important roles in developmental biology and act as cancer biomarkers. Both MMP-2 and MMP-9 contribute to various processes in cancer including invasion [27], metastasis [28], and angiogenesis [29,30]. Moreover, MMP-2 and MMP-9 also contribute to epirubicin resistance in non-small-cell lung cancer [31] and breast cancer [32]. In the present study, we found that ORM1 not only enhanced the proliferation and migration, but also epirubicin resistance of breast cancer cells. Our data also revealed that the expression of MMP-2 and MMP-9 was decreased after downregulating ORM1. In summary, ORM1 promotes the malignant phenotype of breast cancer by upregulating the expression of MMP-2 and MMP-9.

The AKT/ERK pathway is one of the most important signaling pathways by contributing to the inhibition of apoptosis and increasing the proliferation of cancer cells by modulating the activation/inhibition of downstream molecules. AKT/ERK signaling is closely related to the occurrence and development of breast cancer. There have been many studies showing a relationship between the AKT/ERK signaling pathway and cancer. Activated AKT and ERK can inhibit apoptosis, stimulate cell growth, and increase proliferation in many tumors [32–35]. Moreover, Endostar, a recombinant human endostatin, significantly inhibited the metastasis of colon cancer by reducing the phosphorylation of AKT and ERK, and inhibiting the expression of MMP-2 and MMP-9 protein [36]. We found that downregulating ORM1 inhibited the AKT/ERK signaling pathway. Taken together, we demonstrated that ORM1 promotes the malignant phenotype of breast cancer by upregulating the expression of MMP-2 and MMP-9, thus activating the AKT/ERK signaling pathway.

## 5. Conclusion

Our study shows that ORM1 is increased, not only in breast cancer cells, but also in an epirubicin-resistant cell line. Downregulating the expression of ORM1 reversed the malignant phenotype of breast cancer cells by targeting MMP-2 and MMP-9 and activating the AKT/ERK signaling pathway.
